# Prevalence of depression and its correlation with anxiety, headache and sleep disorders among medical staff in the Hainan Province of China

**DOI:** 10.3389/fpubh.2023.1122626

**Published:** 2023-06-27

**Authors:** Guangshuang Lu, Shaobo Xiao, Jiaji He, Wei Xie, Wei Ge, Fanchao Meng, Ying Yang, Shengyuan Yu, Ruozhuo Liu

**Affiliations:** ^1^Department of Pediatrics, The Lu’an Hospital Affiliated to Anhui Medical University, The Lu’an People’s Hospital, Lu’an, China; ^2^Medical School of Chinese PLA, Beijing, China; ^3^Department of Neurology, International Headache Center, The First Medical Center of Chinese PLA General Hospital, Beijing, China

**Keywords:** prevalence, depression, anxiety, headache, sleep disorders (SD), medical staff

## Abstract

**Objective:**

This cross-sectional survey aimed to investigate the prevalence of depression among medical staff and its risk factors as well as the association between depression, anxiety, headache, and sleep disorders.

**Methods:**

Stratified random cluster sampling was used to select medical staff from various departments of four hospitals in Sanya City. The Self-Rating Depression Scale (SDS), Self-Rating Anxiety Scale (SAS), and Pittsburgh Sleep Quality Index (PSQI) were used to quantitatively assess depression, anxiety, and sleep disorders. Correlation and regression analyses were performed to determine factors affecting the depression occurrence and scores.

**Results:**

Among 645 medical staff members, 548 (85%) responded. The 1-year prevalence of depression was 42.7% and the prevalence of depression combined with anxiety, headache, and sleep disorders was 23, 27, and 34.5%, respectively. The prevalence of depression in women, nurses, the unmarried or single group, and the rotating-shift population was significantly higher than that in men (48.3% vs. 27.1%, odds ratio OR = 2.512), doctors (55.2% vs. 26.7%, OR = 3.388), the married group (50.5% vs. 35.8%, OR = 1.900), and the day-shift population (35.2% vs. 7.5%, OR = 1.719). The occurrence of depression was correlated with anxiety, sleep disorders, headache, and migraines, with anxiety having the highest correlation (Spearman’s Rho = 0.531). The SDS was significantly correlated with the SAS and PSQI (Spearman’s Rho = 0.801, 0.503) and was also related to the presence of headache and migraine (Spearman Rho = 0.228, 0.159). Multiple logistic regression indicated that nurse occupation and anxiety were risk factors for depression, while grades of anxiety, sleep disorders and nurse occupation were risk factors for the degree of depression in multiple linear regression.

**Conclusion:**

The prevalence of depression among medical staff was higher than that in the general population, especially among women, nurses, unmarried people, and rotating-shift workers. Depression is associated with anxiety, sleep disorders, headache, and migraines. Anxiety and nursing occupation are risk factors for depression. This study provides a reference for the promotion of occupational health among medical professionals.

## 1. Introduction

The 2019 global burden of diseases study reported that depression ranked second in years lived with disability and headache disorders ranked third among all age groups and both sexes (1, 2). The global incidence of depression is rapidly increasing. The World Health Organization predicts that depression will become a primary disease burden by 2030.

A survey of adults with mental disorders covering 31 provinces and cities in China from 2013 to 2015 showed that the lifetime prevalence of depressive disorders in Chinese adults was 6.8%; however, only 0.5% of patients received adequate treatment (3). Faced with high-intensity and demanding work, medical staff often suffer from depression, anxiety, headache, and sleep disorders, which not only cause pain to individuals and families but also affect work efficiency, social productivity, and cause social burden. A meta-analysis of clinical studies and population surveys from 2000 to 2020 suggested that the primary headache comorbidities included depression, hypertension, anxiety, diabetes, and sleep disorders (4). Thus, depression, anxiety, headache, and sleep disorders are highly prevalent and often interact with each other but are not fully recognized or treated.

Our research team conducted an epidemiological survey on the prevalence of primary headache among medical staff in Sanya city, Hainan province, and found that the prevalence of primary headache among medical staff was higher than that among the general population (5). Based on a previous investigation, we added depression- and anxiety-related score data and investigated the correlation between depression, anxiety, headache, sleep disorders, and the risk factors for depression to provide data for improving the health status of medical staff.

## 2. Materials and methods

### 2.1. Survey methods

This is a cross-sectional study. The research protocol was approved by the Ethics Committee of the Chinese PLA General Hospital. All patients were informed of the purpose of the study and provided informed consent prior to participation.

The survey subjects were medical staff who were randomly selected from three tertiary hospitals (Hainan Hospital of Chinese PLA General Hospital, Third People’s Hospital of Hainan Province, and People’s Hospital of Sanya) and one secondary hospital (Chinese PLA No. 425 Hospital) in Sanya, South China, from May 2018 to October 2018. After a preliminary survey of one department in each hospital, stratified random cluster sampling was performed using epidemiological methods. The departments in all hospitals were divided into three groups: internal medicine, surgery, and others (Emergency Department and Radiology Department), and eight clinical departments were randomly selected. Physicians and nurses were selected as a whole group for this investigation. The survey was divided into two parts: a questionnaire and an interview (face-to-face and telephone). All participants who reported depression, anxiety, headache, or sleep disorders were interviewed by neurological and psychiatric specialists after reviewing their questionnaires. The notes associated with the questionnaire were explained and the participants’ questions were answered in reference to the Diagnostic and Statistical Manual of Mental Disorders, Fifth Edition and the International Classification of Headache Disorders, Third Edition criteria. Each participant answered the structured questionnaires, and data were collected on demographics, occupation-related factors, and characteristics such as depression, anxiety, headache, and sleep disturbance over the past year.

### 2.2. The questionnaire

The questionnaire consists of four parts.

- Demographic data and medical professional characteristics included sex, age, marital status (single or divorced, married), body mass index (BMI, graded as underweight, normal weight, overweight, obese), educational background (college or lower, bachelor’s degree, master’s degree, or above), occupation (doctor or nurse), work seniority, professional titles (junior, senior, or advanced), work arrangements (day-shift or rotating shift), and number of night shifts. For this part, we referred to the results of our previous survey (5).
- The Self-Rating Depression Scale (SDS) and the Self-Rating Anxiety Scale (SAS) included 20 items that measured mental, physical, and emotional symptoms and were rated by respondents in terms of how often the symptoms were experienced over the past week, using a 4-point scale ranging from 1 (none or very little) to 4 (most or all of the time). The raw score was calculated and converted into a standard score. The SDS scores were interpreted as follows: 53–62, mild depression symptoms; 63–72, mild, moderate depression symptoms; and > 72, severe depression symptoms. SAS scores of 50–59 were classified as mild anxiety, 60–69 as moderate anxiety, and > 69 as severe anxiety. Both scoring systems have been shown to have good internal consistency, and the Cronbach alpha coefficients of the two scores were 0.81 and 0.82, respectively (6). Wang, Cai, and Xu (7) suggested that an index score of 53 (raw score 42) for depression was more appropriate for Chinese populations and this score has since been widely adopted (8, 9). At this score sensitivity is 96.5% and specificity 59.3% according to the Dunstan and Scott’s research (10).
- The headache profile section (25 questions) included headache nature, headache degree (visual analogue scale, VAS), headache frequency, accompanying symptoms, and aggravating and alleviating factors. The reliability and validity of the Chinese version of the questionnaire for headache diagnosis has been tested in China and used in epidemiological investigations of nationwide demographic headache (11–13). VAS scores have been shown to have high reliability, with an intraclass correlation coefficient of 0.97 (14). In this questionnaire, headache disorders included migraine, tension-type headache (TTH), and other types such as neuralgia, chronic daily headache, and unclassified headaches. Trigeminal autonomic cephalalgias, other primary headache disorders, and secondary headaches were not included.
- The Pittsburg Sleep Quality Index (PSQI) uses 18 self-rated items to evaluate seven dimensions of sleep. Each dimension is scored on a scale of 0 to 3, and the total PSQI score for each dimension ranges from 0 to 21. The PSQI scores ranged from 0–5 for normal sleep, 6–10 for mild sleep disorders, 11–15 for moderate sleep disorders, and 16–21 for severe sleep disorders. In most studies, the Cronbach’s alpha coefficient of the PSQI score was between 0.70 and 0.83, showing good internal consistency (15).

### 2.3. Statistical analysis

Epidata 3.1 was used for data entry, and statistical analyses were performed using SPSS version 26.0. The Kolmogorov–Smirnov test was used to examine the normality of the distribution of continuous variables. Data are expressed as mean (standard deviation) for normally distributed variables or median (interquartile range) for non-normally distributed variables, and categorical variables are summarized as the number and percentage.

Depression, anxiety, headache, and sleep disorders were defined as binary variables. Two subcategories of headache, migraines, and TTH were used as variables. The Chi-square test (categorical variables) and the Mann–Whitney U test (quantitative variables with non-normal distribution) were applied to compared the demographic information of the two groups of individuals with or without depression. Because there were ordinal categorical variables such as professional titles in the demographic data, we used a nonparametric Spearman correlation analysis for depression occurrence (binary variable). Considering that SDS scores are continuous variables that do not conform to a normal distribution, Spearman correlation analysis was also used, and Spearman’s correlation coefficient (Spearman’s rho) was calculated. The statistical significance level was set at *p* < 0.05.

Univariate logistic regression analysis was used to identify odds ratios (OR) and 95% confidence intervals according to demographic and occupational characteristics, anxiety, headache type, and sleep disorders. The independent variables with a *p*-value <0.10 by univariate logistic regression screening were included in the candidate variables of the multiple logistic regression model to predict the occurrence of depression. And multiple linear regression was also used to predict depression SDS scores.

## 3. Results

A total of 645 medical staff members (280 physicians and 365 nurses) were invited to participate in the study, of whom 22 refused to complete the survey. Another 75 participants either did not answer the questionnaire completely or did not meet the response requirements, with a correct response rate of 85%. A total of 548 respondents (240 doctors and 308 nurses) completed the survey. There were 144 men and 404 women, aged between 20 and 60 years, with a mean age of 30.5 ± 7.2. Of the 240 doctors, 138 were men and 102 were women. There were 6 men and 302 women among the 308 nurses.

### 3.1. Prevalence of depression, anxiety, headache, and sleep disorders

**Figure 1:**
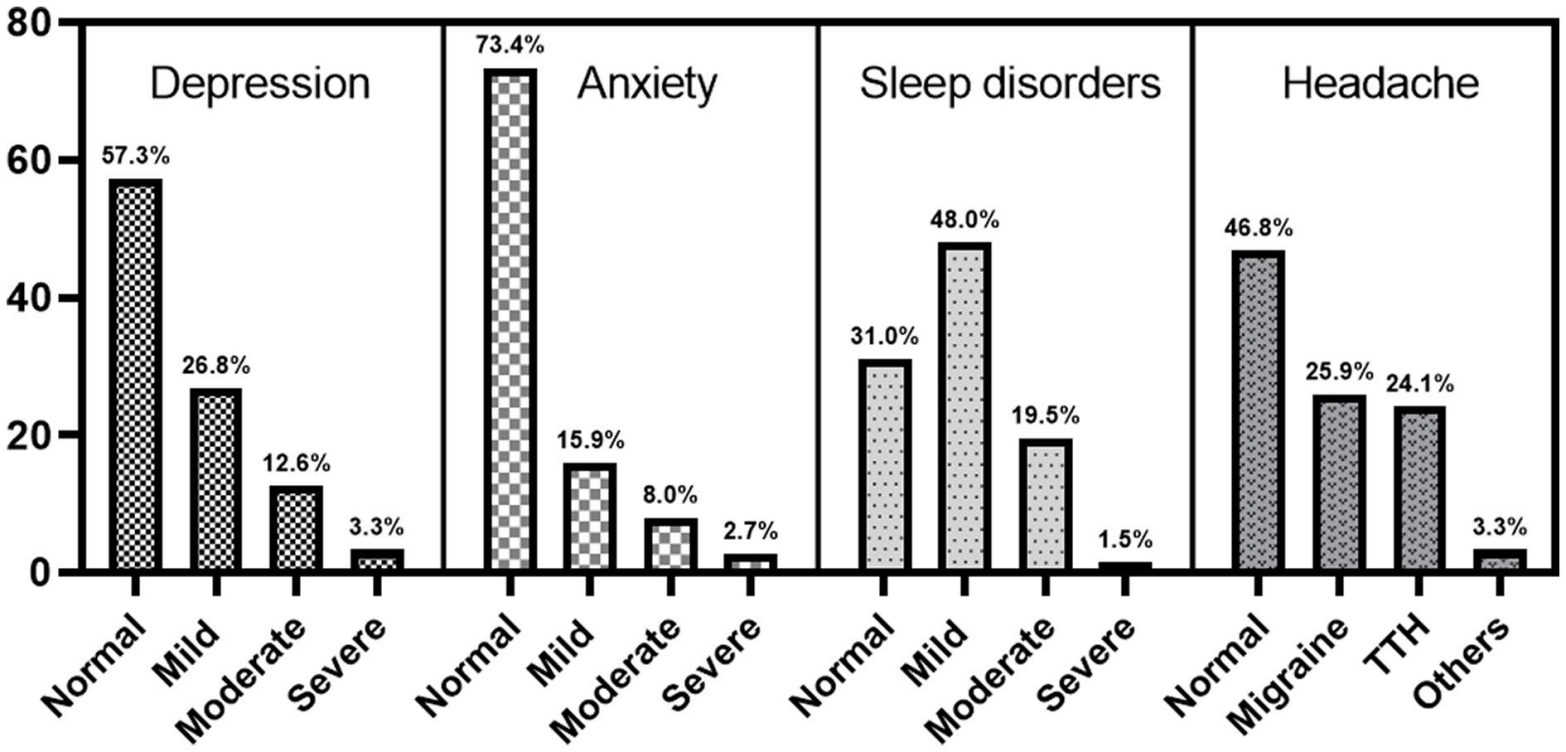
Prevalence of depression, anxiety, headache, and sleep disorders among 548 medical staff.

**Figure 2:**
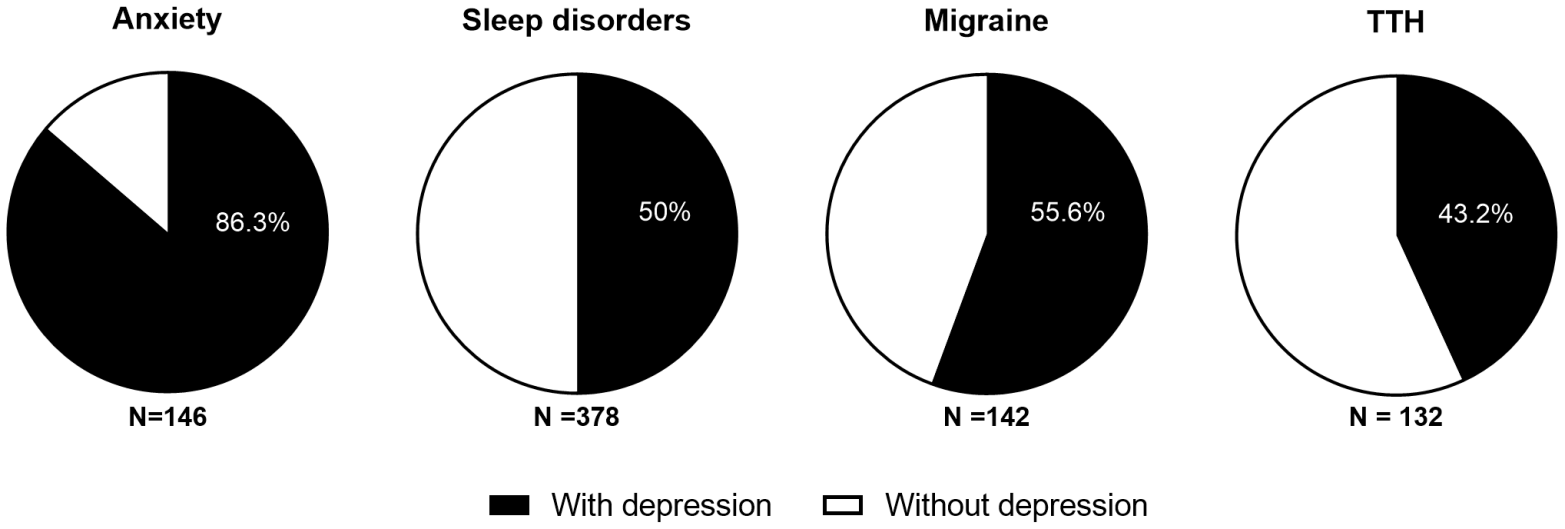
Proportion of patients with depression in anxiety, sleep disorder, migraine, and TTH.

Among the 548 medical staff members, the 1-year prevalence of depression was 42.7% (mild, 26.8%; moderate and severe, 15.9%), the prevalence of headache disorders was 53.3% (migraine, 25.9%; TTH, 24.1%; other types, 3.3%), and the prevalence of anxiety and sleep disorders was 26.6% and 69.0%, respectively (see Figure 1). Among them, the prevalence of depression combined with anxiety, headache, and sleep disorders was 23, 27% (migraine 14.4%, TTH 10.4%), and 34.5%, respectively; the proportions of patients with depression with anxiety, sleep disorder, migraine, and TTH were 86.3%, 50%, 55.6%, and 43.2%, respectively (see Figure 2), but only 11 respondents (4.7%) had received psychiatric medication for depression.

We found that the prevalence of depression in women was significantly higher than in men (48.3% vs. 27.1%, OR = 2.512), and women were approximately five times as likely to have depression as males were (35.6% vs. 7.1%). The median age in the depressed group (28 years) was lower than that in the non-depressed group (30 years). The prevalence of depression in unmarried or divorced individuals was significantly higher than that in married individuals (50.5% vs. 35.8%, OR = 1.900). The prevalence of depression among nurses was nearly twice as high as that among doctors (55.2% vs. 26.7%, OR = 3.388), and was significantly higher among rotating-shift workers than among day-shift workers (35.2% vs. 7.5%, OR = 1.719). The total prevalence of overweight and obesity in the medical population was approximately 33.1%, among which 10.6% were depressed and 32.1% were depressed in the population with a BMI <23 kg/m^2^. In addition, with improvements in educational backgrounds and professional titles, the prevalence of depression gradually decreased.

The prevalence of mild, moderate, and severe anxiety was higher in patients with depression than in those without. The prevalence of migraine was higher in the depressive group (55.6% vs. 44.4%, OR = 2.031), whereas the prevalence of TTH was higher in the non-depressive group (43.2% vs. 56.8%). In comparison with the sleep disorder group, we found that the prevalence of mild sleep disorders was similar in the two groups, while the prevalence of moderate and severe sleep disorders was higher in the depressive group. Table 1 presents the results of the study.

**Table 1:** Comparison of the characteristics of medical staff with and without depression.

		With depression (*n* = 234)	Without depression (*n* = 314)	*P*-value	OR (95%Cl)
Sex (*n*, %)	Male	39 (27.1%)	105 (72.9%)	<0.001^a^	0.398 (0.263–0.604)
	Female	195 (48.3%)	209 (51.7%)		
Age	Mean (±SD)	28.8 (±5.7)	31.7 (±8.0)	<0.001^b^	1.833 (1.302–2.582)
Marital status	Single or divorced	129 (51.2%)	126 (48.8%)	<0.001^a^	1.900 (1.348–2.678)
	Married	105 (35.6%)	188 (64.4%)		
BMI (kg/m^2^)	Underweight <18.5	51 (56.0%)	40 (44.0%)	0.001^a^	
	Normal weight 18.5–22.9	125 (45.5%)	150 (54.5%)		
	Overweight 23–25	26 (31.3%)	57 (68.7%)		
	Obese ≥25	32 (32.3%)	67 (67.7%)		
Occupation	Doctor	64 (26.7%)	176 (73.3%)	<0.001^a^	0.295 (0.205–0.425)
	Nurse	170 (55.2%)	138 (44.8%)		
Educational background	College or lower	101 (54.6%)	84 (45.4%)	<0.001^a^	1.716 (1.186–2.483)
	Bachelor	124 (41.2%)	177 (58.8%)		4.126 (1.963–8.673)
	Masters or above	9 (14.5%)	53 (85.5%)		
Work Seniority (years)	Median (P25, P75)	5 (3,8)	6 (3,12)	0.021^b^	
Work arrangements	Dayshift	41 (32.8%)	84 (67.2%)	0.011^a^	0.582 (0.382–0.885)
	Rotating shift	193 (45.6%)	230 (54.4%)		
Night shifts	Median (P25, P75)	5 (4,8)	5 (0,7)	0.003^b^	
Professional titles	Junior	199 (48.8%)	209 (51.2%)	<0.001^a^	2.698 (1.629–4.467)
	Senior	24 (26.1%)	68 (73.9%)		1.187 (0.524–2.691)
	Advanced	11 (8.8%)	37 (77.1%)		
Anxiety	Total	126 (86.3%)	20 (13.7%)	<0.001^a^	17.150 (10.188–28.869)
	Mild	69 (79.3%)	18 (20.7%)		
	Moderate	42 (95.5%)	2 (4.5%)		
	Severe	15 (100%)	0		
Headache	Total	148 (50.7%)	144 (49.3%)	<0.001^a^	
	Migraine	79 (55.6%)	63 (44.4%)		2.032 (1.437–2.872)
	Tension-type headache	57 (43.2%)	75 (56.8%)		
Sleep disorders	Total	189 (50.0%)	189 (50.0%)	<0.001^a^	
	Mild	104 (39.5%)	159 (60.5%)		2.778 (1.870–4.127)
	Moderate	77 (72.0%)	30 (28.0%)		
	Severe	8 (100%)	0		

a Chi-squared test.

b Mann-Whitney U test.

OR, odds ratio; CI, confidence interval; SD, standard deviation; BMI, body mass index.

### 3.2. Correlation analysis and multiple logistic regression of the occurrence of depression

**Figure 3:**
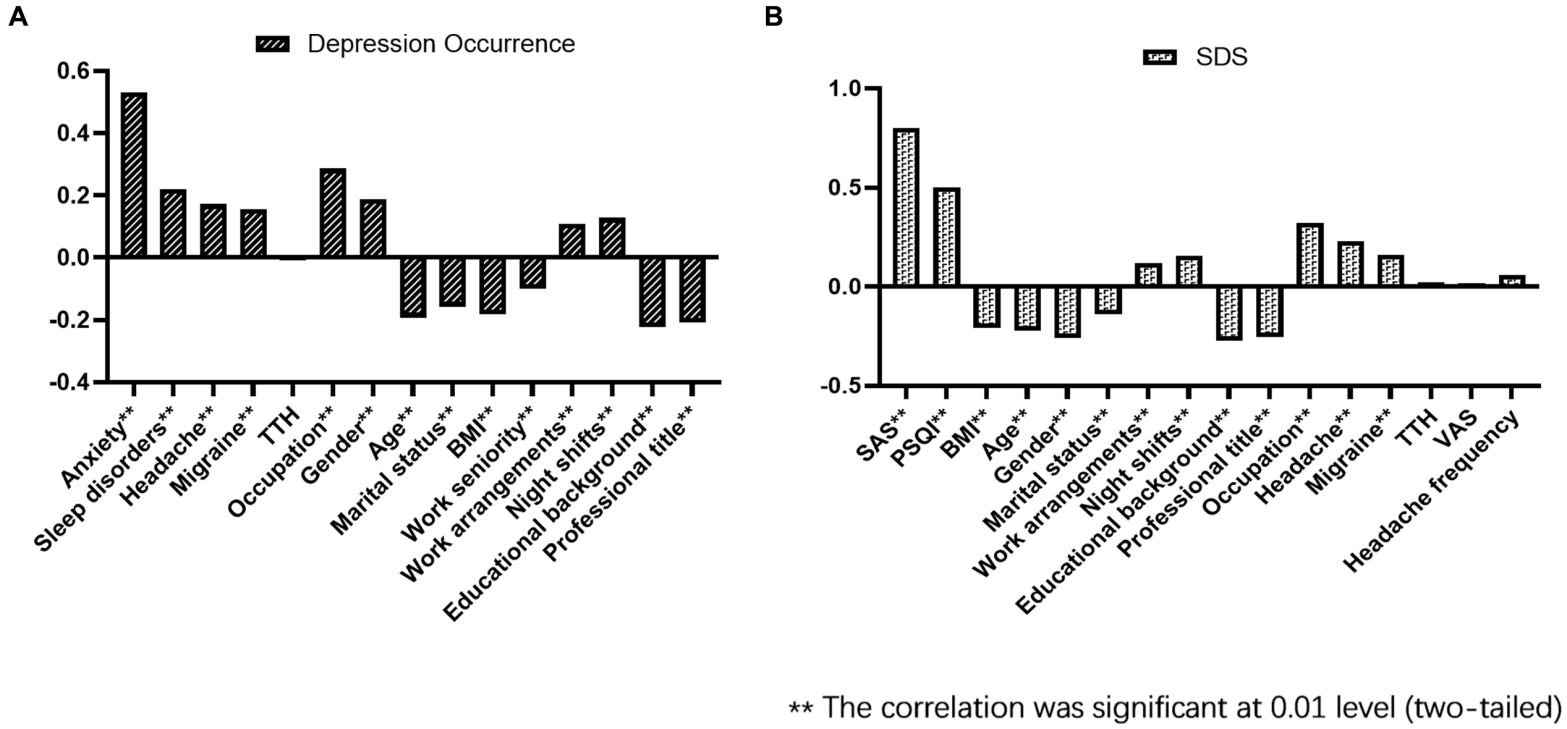
Nonparametric Spearman correlation analysis of depression occurrence **(A)** and SDS **(B)**.

We first used nonparametric Spearman correlation analysis of the binary classification variables of depression and anxiety, headache, and sleep disorders, and then examined their association with demographic characteristic factors. The results showed that depression was associated with anxiety, sleep disorders, total headache, and migraines, with the largest correlation coefficient for anxiety (Spearman’s rho = 0.531). However, no significant association was observed between TTH levels and depression. Other related factors included age, sex, BMI, professional title, educational background, marital status, occupation, work arrangements, and night shifts (Figure 3A).

**Figure 4:**
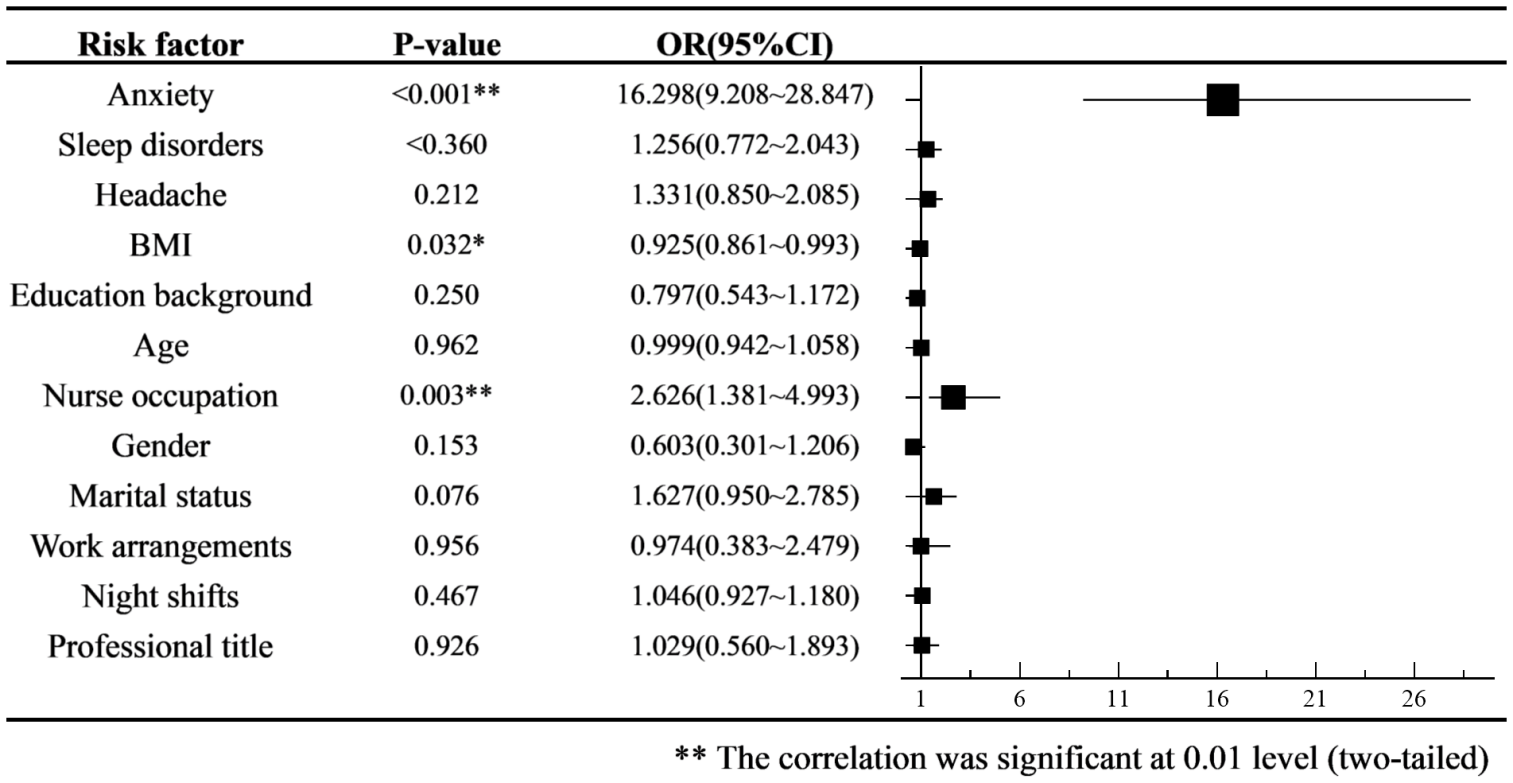
Odds ratio (ORs) forest plot of multiple logistic regression for depression occurrence.

We performed a multifactor binary logistic regression analysis of the occurrence of depression. The TTH, headache frequency, and VAS scores for headache intensity were found have *p*-values >0.1 as determined by univariate logistic regression; therefore, they were not included in the multifactor regression model. The results showed that anxiety and nursing occupation were risk factors, whereas BMI was a protective factor against depression (Figure 4). The predictive accuracy of the multifactor binary logistic regression model was 78.5%.

### 3.3. Correlation analysis and multiple linear regression of depression SDS scores

We further analyzed the correlation between SDS scores for depression severity and the quantitative variables of SAS, PSQI, headache frequency, headache intensity, VAS scores, and demographic data. The results showed that the severity of depression was significantly correlated with the grades of anxiety and sleep disorders (Spearman’s rho = 0.801, 0.503) and the presence or absence of headaches and migraines. However, there was no significant correlation between SDS scores and headache frequency, headache intensity, or TTH; however, headache frequency and headache intensity affected the PSQI score of sleep disorders (Spearman’s rho = 0.205, 0.166, *p* < 0.001). In addition, the results showed that the severity of depression was positively correlated with nurse occupation, night shifts, and working arrangements, and negatively correlated with age, sex, marital status, BMI, educational background, and professional titles (see Figure 3B).

**Figure 5:**
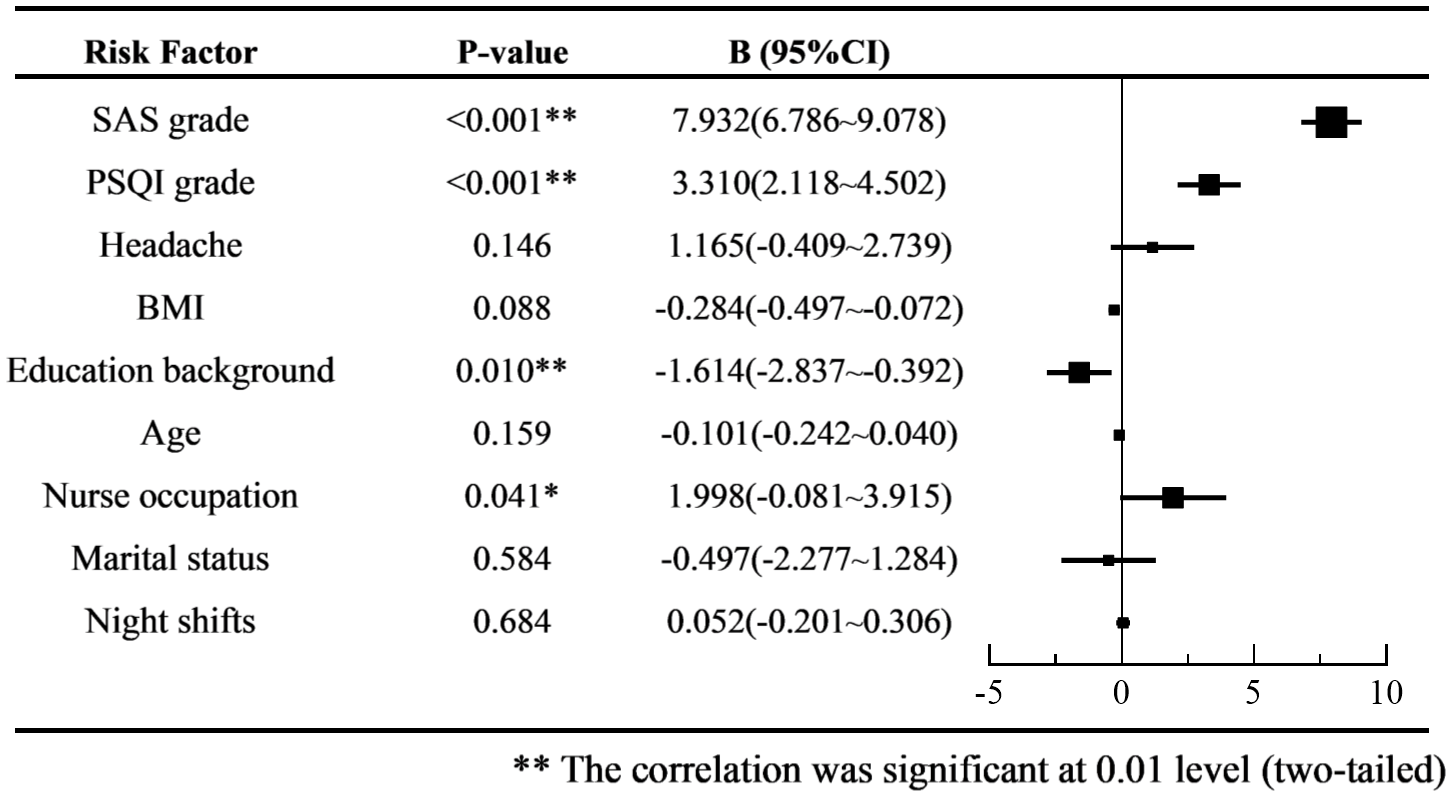
Forest plot of multiple linear regression coefficient B of SDS score.

We then performed a multiple linear regression analysis of the SDS scores to predict depression severity. The VAS scores for headache intensity were found to have a *p*-value >0.1 in the early screening process; therefore, they were not included in the regression model. Considering the multicollinearity between age, working years, professional title, medical profession, sex, working arrangement, and night shift frequency, we only included variables such as age, nurse occupation, and night shift frequency. According to the results of the multiple linear regression analysis, grades of anxiety, sleep disorders, and nursing occupation were risk factors, while educational background was a protective factor against depression severity (see Figure 5).

## 4. Discussion

### 4.1. Prevalence

The prevalence of depression in the medical population was significantly higher than that in the general population, particularly among women and nurses. The estimated prevalence of depression among resident doctors in a systematic review published in 2015 was 28.8%, ranging from 20.9 to 43.2%, depending on the assessment tools used (16). Many studies have reported that the prevalence of depression among nurses ranges from 18 to 64.8% (17–21), and some studies have suggested that the prevalence of depression among nurses is nearly twice that among other professional individuals (22). The prevalence of depressive symptoms among Chinese nurses between 1996 and 2019 was approximately 43.8% (23). In our study, the prevalence of depression among physicians and nurses was 26.7% and 55.2%, respectively, which is consistent with the results of previous reports and indicates that the mental health of nurses requires more attention from society.

### 4.2. Anxiety and depression

The occurrence of depression was correlated with anxiety, sleep disorders, headache, and migraine, among which the correlation with anxiety was the strongest. Previous studies have consistently identified risk factors for depression, including cognitive and cognitive processes, stressors, sociodemographic factors (e.g., being female), parental depression, and certain behaviors and personalities. Stress is a risk factor for depression, and more than 80% of the individuals in the community sample who met the criteria for clinically significant depression had experienced a recent major life event or persistent stressor. These risks and depression interact in a bidirectional and dynamic manner (24). Medical staff face multiple stressors from patients, society, family, and individuals, which often leads to anxiety. Anxiety disorders usually first appear in early childhood and adolescence, long before depression develops, and 56% of individuals with anxiety disorders develop depression. Anxiety disorders can be described as risk factors for secondary depression in this age group (25). Our study found that anxiety was significantly correlated with the occurrence and severity of depression, and that stress may be the dominant factor. Changes in neurobiological substrates related to stress regulation and maladjustment of transmitter activity are possible mechanisms underlying anxiety and depression (26). We also found that the higher the educational level, the lower the prevalence and severity of depression. This is because people with different degrees have different levels of self-regulation and cognitive abilities to cope with stress, and many studies have shown that highly educated adults have lower levels of depression. Therefore, we believe that there is a cognition-stress-anxiety-depression relationship.

### 4.3. Headache and depression

Our survey found that the occurrence of depression was associated with the presence or absence of headache or migraine, but there was no significant correlation between the severity of depression and the severity score of headache, which is consistent with previous reports (27). There was a bidirectional association between headache and depression, with patients with depression having an increased risk of migraine, and migraineurs having an increased risk of depression. Moreover, the symptoms of migraine and depression are more severe than those who suffer alone (28, 29). Headache frequency has also been reported to affect depression scores (30). In our study, no significant difference in TTH was found between groups with and without depression, which differs from the results of a general population sample (31). There is little epidemiological data on TTH among medical staff. The reasons for this difference are related to the characteristics and working modes of medical staff, among whom TTH is more likely to be induced by long-term desk work and frequent night shifts.

### 4.4. Sleep disorders and depression

In our survey, sleep disorders were significantly associated with the occurrence and severity of depression. It is currently believed that the relationship between depression and sleep disorders is reciprocal. A longitudinal study showed that both anxiety and depression are significantly linked to the incidence, but not persistence, of sleep disorders (32). Studies have also identified sleep disorders as an independent risk factor for the onset or recurrence of depression in young, middle-aged, and older adults (33, 34). Moreover, one of the symptoms most consistent with major depressive disorder is sleep disturbance (35). In addition, patients with depression and sleep disorders tended to have more severe depressive symptoms, longer treatment times, and lower remission rates (36). Therefore, more attention should be paid to sleep disorder interventions when treating depression.

### 4.5. BMI and depression

There have been many studies on the relationship between depression and BMI, but consistency has been poor. There is a range from negative correlation studies (37–41) on “happy obesity” to positive correlation studies (42, 43), and there are also studies showing that the correlation between BMI and depression can be ignored (44). Our results showed that BMI was negatively associated with depression, although the correlation coefficient was small. The reason for this may be that different ages, sexes, and educational backgrounds have different body shape cognitions. Further stratified research on the relationship between BMI and depression is required.

### 4.6. Limitations

In terms of limitations, our study relied on the participants’ memories, and recall bias may have affected the reliability of the survey data. Moreover, only one assessment tool was used for each disease, which is another limitation of this study. Multiple assessment tools and objective examinations can be combined to refine the stressors in the medical population for further analysis. Further, as a cross-sectional study, we found associations between depression, anxiety, headache, and sleep disorders, but could not disentangle the cause and effect. It is well known that genetic factors play an important role in the development of depression, but family history of depression and other factors such as alcohol use and sun exposure time were not included in our survey, which should be explored in future studies.

## 5. Conclusion

Overall, emotional disorders such as depression are more prevalent in the healthcare population, especially among women and nurses, but they rarely attract attention and lack effective treatment. Depression, anxiety, headache, and sleep disorders often co-occur and interact with each other. Assessment of mood, headache, and sleep problems is of great significance for more effective prevention and treatment of depression among medical staff.

## Data availability statement

The raw data supporting the conclusions of this article will be made available by the authors, without undue reservation.

## Ethics statement

This study was approved by the ethics committee of the Chinese PLA General Hospital. The patients/participants provided their written informed consent to participate in this study.

## Author contributions

GL was responsible for reviewing the literature and writing the manuscript. SX was responsible for the data analysis. WX and WG provided the raw data and guidance. JH, FM, and YY were responsible for issuing and recalling the SDS/SAS/PSQI questionnaires. RL and SY were the principal investigators responsible for the study design, data analysis and interpretation, manuscript revision, had full access to all data in the study, and had the final responsibility for the decision to submit the manuscript for publication. All authors contributed to the article and approved the submitted version.

## Funding

This study received funding from the Chinese PLA General Hospital 2019, Military Medicine Transformation Project (grant ZH19002), and 2020 Equipment Research and Development Project (grant LB2020LA060003).

## Conflict of interest

The authors declare that the research was conducted in the absence of any commercial or financial relationships that could be construed as a potential conflict of interest.

## Publisher’s note

All claims expressed in this article are solely those of the authors and do not necessarily represent those of their affiliated organizations, or those of the publisher, the editors and the reviewers. Any product that may be evaluated in this article, or claim that may be made by its manufacturer, is not guaranteed or endorsed by the publisher.

## Abbreviations

BMI: body mass index
OR: odds ratio
PSQI: pittsburgh sleep quality index
SAS: self-rating anxiety scale
SDS: self-rating depression scale
TTH: tension-type headache
VAS: visual analog score

## References

[1] Vos T Lim SS Abbafati C Abbas KM Abbasi M Abbasifard M. Global burden of 369 diseases and injuries in 204 countries and territories, 1990-2019: a systematic analysis for the global burden of disease study 2019. Lancet. (2020) 396:1204–22. doi: 10.1016/S0140-6736(20)30925-9 33069326 PMC7567026

[2] Steiner TJ Stovner LJ Jensen R Uluduz D Katsarava Z. Migraine remains second among the world's causes of disability, and first among young women: findings from GBD2019. J Headache Pain. (2020) 21:137. doi: 10.1186/s10194-020-01208-0, PMID: 33267788 PMC7708887

[3] Lu J Xu X Huang Y Li T Ma C Xu G. Prevalence of depressive disorders and treatment in China: a cross-sectional epidemiological study. Lancet Psychiatry. (2021) 8:981–90. doi: 10.1016/S2215-0366(21)00251-0, PMID: 34559991

[4] Caponnetto V Deodato M Robotti M Koutsokera M Pozzilli V Galati C. Comorbidities of primary headache disorders: a literature review with meta-analysis. J Headache Pain. (2021) 22:71. doi: 10.1186/s10194-021-01281-z, PMID: 34261435 PMC8278743

[5] Xie W Li R He M Cui F Sun T Xiong J. Prevalence and risk factors associated with headache amongst medical staff in South China. J Headache Pain. (2020) 21:5. doi: 10.1186/s10194-020-1075-z, PMID: 31937239 PMC6961346

[6] Tanaka-Matsumi J Kameoka VA. Reliabilities and concurrent validities of popular self-report measures of depression, anxiety, and social desirability. J Consult Clin Psychol. (1986) 54:328–33. doi: 10.1037/0022-006X.54.3.328, PMID: 3722561

[7] Wang CF Cai ZH Xu Q. Evaluation analysis of self-rating disorder scale in 1,340 people. Chin J Nervous Mental Dis. (1986) 12:267–8.

[8] Zhang J Yin Y Wen Y Shi F Wang J. Anxiety and depression in patients with pulmonary arterial hypertension in Northwest China: a cross-sectional study. Front Psych. (2021) 12:758120. doi: 10.3389/fpsyt.2021.758120 PMC8854771 35185632

[9] Zhang XR Huang QM Wang XM Cheng X Li ZH Wang ZH. Prevalence of anxiety and depression symptoms, and association with epidemic-related factors during the epidemic period of COVID-19 among 123,768 workers in China: a large cross-sectional study. J Affect Disord. (2020) 277:495–502. doi: 10.1016/j.jad.2020.08.041, PMID: 32882506 PMC7448744

[10] Dunstan DA Scott N. Clarification of the cut-off score for Zung's self-rating depression scale. BMC Psychiatry. (2019) 19:177. doi: 10.1186/s12888-019-2161-0, PMID: 31185948 PMC6558728

[11] Wang Y Xie J Yang F Wu S Wang H Zhang X. The prevalence of primary headache disorders and their associated factors among nursing staff in North China. J Headache Pain. (2015) 16:4. doi: 10.1186/1129-2377-16-4, PMID: 25582043 PMC4405508

[12] Yu S Liu R Zhao G Yang X Qiao X Feng J. The prevalence and burden of primary headaches in China: a population-based door-to-door survey. Headache. (2012) 52:582–91. doi: 10.1111/j.1526-4610.2011.02061.x 22590713

[13] Yu SY Cao XT Zhao G Yang XS Qiao XY Fang YN. The burden of headache in China: validation of diagnostic questionnaire for a population-based survey. J Headache Pain. (2011) 12:141–6. doi: 10.1007/s10194-011-0336-2, PMID: 21452008 PMC3072517

[14] Bijur PE Silver W Gallagher EJ. Reliability of the visual analog scale for measurement of acute pain. Acad Emerg Med. (2001) 8:1153–7. doi: 10.1111/j.1553-2712.2001.tb01132.x 11733293

[15] Mollayeva T Thurairajah P Burton K Mollayeva S Shapiro CM Colantonio A. The Pittsburgh sleep quality index as a screening tool for sleep dysfunction in clinical and non-clinical samples: a systematic review and meta-analysis. Sleep Med Rev. (2016) 25:52–73. doi: 10.1016/j.smrv.2015.01.009, PMID: 26163057

[16] Mata DA Ramos MA Bansal N Khan R Guille C Di Angelantonio E. Prevalence of depression and depressive symptoms among resident physicians: a systematic review and meta-analysis. JAMA. (2015) 314:2373–83. doi: 10.1001/jama.2015.15845, PMID: 26647259 PMC4866499

[17] Jeong YM Min A. Depression, help-seeking attitude, sleep quality, and missed nursing care among nurses in Korean hospitals: a cross-sectional study. J Nurs Scholarsh. (2022) 54:135. doi: 10.1111/jnu.12647 33666354

[18] Cheung T Yip PS. Lifestyle and depression among Hong Kong nurses. Int J Environ Res Public Health. (2016) 13:135. doi: 10.3390/ijerph13010135, PMID: 26784216 PMC4730526

[19] Zhang Y Duffy JF De Castillero ER. Do sleep disturbances mediate the association between work-family conflict and depressive symptoms among nurses? A cross-sectional study. J Psychiatr Ment Health Nurs. (2017) 24:620–8. doi: 10.1111/jpm.12409, PMID: 28635074 PMC5585039

[20] Lee HY Kim MS Kim O Lee IH Kim HK. Association between shift work and severity of depressive symptoms among female nurses: the Korea Nurses' health study. J Nurs Manag. (2016) 24:192–200. doi: 10.1111/jonm.12298, PMID: 25950801

[21] Chang Y Wang PC Li HH Liu YC. Relations among depression, self-efficacy and optimism in a sample of nurses in Taiwan. J Nurs Manag. (2011) 19:769–76. doi: 10.1111/j.1365-2834.2010.01180.x, PMID: 21899630

[22] Letvak S Ruhm CJ McCoy T. Depression in hospital-employed nurses. Clin Nurse Spec. (2012) 26:177–82. doi: 10.1097/NUR.0b013e3182503ef0, PMID: 22504476

[23] Xie N Qin Y Wang T Zeng Y Deng X Guan L. Prevalence of depressive symptoms among nurses in China: a systematic review and meta-analysis. PLoS One. (2020) 15:e0235448. doi: 10.1371/journal.pone.0235448, PMID: 32634150 PMC7340293

[24] Hammen C. Risk factors for depression: an autobiographical review. Annu Rev Clin Psychol. (2018) 14:1–28. doi: 10.1146/annurev-clinpsy-050817-084811, PMID: 29328780

[25] Wittchen HU Kessler RC Pfister H Höfler M Lieb RJ. Why do people with anxiety disorders become depressed? A prospective-longitudinal community study. Acta Psychiatr Scand Suppl. (2000) 102:14–23. doi: 10.1111/j.0065-1591.2000.acp29-03.x 11131466

[26] McEwen BS. The neurobiology of stress: from serendipity to clinical relevance. Brain Res. (2000) 886:172–89. doi: 10.1016/S0006-8993(00)02950-4, PMID: 11119695

[27] Lee DH Kim KM Cho SJ Kim WJ Yang KI Yun CH. Impacts of migraine on the prevalence and clinical presentation of depression: a population-based study. J Affect Disord. (2020) 272:215–22. doi: 10.1016/j.jad.2020.03.102, PMID: 32553361

[28] Victor TW Hu X Campbell J White RE Buse DC Lipton RB. Association between migraine, anxiety and depression. Cephalalgia. (2010) 30:567–75. doi: 10.1111/j.1468-2982.2009.01944.x 19614684

[29] Song TJ Cho SJ Kim WJ Yang KI Yun CH Chu MK. Anxiety and depression in probable migraine: a population-based study. Cephalalgia. (2017) 37:845–54. doi: 10.1177/0333102416653235, PMID: 27250234

[30] Ashina S Bendtsen L Buse DC Lyngberg AC Lipton RB Jensen R. Neuroticism, depression and pain perception in migraine and tension-type headache. Acta Neurol Scand. (2017) 136:470–6. doi: 10.1111/ane.12751, PMID: 28261782

[31] Song TJ Cho SJ Kim WJ Yang KI Yun CH Chu MK. Anxiety and depression in tension-type headache: a population-based study. PLoS One. (2016) 11:e0165316. doi: 10.1371/journal.pone.0165316, PMID: 27783660 PMC5082613

[32] Morphy H Dunn KM Lewis M Boardman HF Croft PR. Epidemiology of insomnia: a longitudinal study in a UK population. Sleep. (2007) 30:274–80. doi: 10.1093/sleep/30.3.274 PMID: 17425223

[33] Baglioni C Battagliese G Feige B Spiegelhalder K Nissen C Voderholzer U. Insomnia as a predictor of depression: a meta-analytic evaluation of longitudinal epidemiological studies. J Affect Disord. (2011) 135:10–9. doi: 10.1016/j.jad.2011.01.011, PMID: 21300408

[34] Chang PP Ford DE Mead LA Cooper-Patrick L Klag MJ. Insomnia in young men and subsequent depression. The Johns Hopkins precursors study. Am J Epidemiol. (1997) 146:105–14. doi: 10.1093/oxfordjournals.aje.a009241, PMID: 9230772

[35] Peterson MJ Benca RM. Sleep in mood disorders. Psychiatr Clin North Am. (2006) 29:1009–32. doi: 10.1016/j.psc.2006.09.003 17118279

[36] Fang H Tu S Sheng J Shao A. Depression in sleep disturbance: a review on a bidirectional relationship, mechanisms and treatment. J Cell Mol Med. (2019) 23:2324–32. doi: 10.1111/jcmm.14170, PMID: 30734486 PMC6433686

[37] Crisp AH McGuiness B. Jolly fat: relation between obesity and psychoneurosis in general population. Br Med J. (1976) 1:7–9. doi: 10.1136/bmj.1.6000.7, PMID: 1247732 PMC1638245

[38] Bin Li Z Yin Ho S Man Chan W Sang Ho K Pik Li M Leung GM. Obesity and depressive symptoms in Chinese elderly. Int J Geriatr Psychiatry. (2004) 19:68–74. doi: 10.1002/gps.1040 14716701

[39] Kuriyama S Koizumi Y Matsuda-Ohmori K Seki T Shimazu T Hozawa A. Obesity and depressive symptoms in elderly Japanese: the Tsurugaya project. J Psychosom Res. (2006) 60:229–35. doi: 10.1016/j.jpsychores.2005.07.010, PMID: 16516653

[40] Chang HH Yen ST. Association between obesity and depression: evidence from a longitudinal sample of the elderly in Taiwan. Aging Ment Health. (2012) 16:173–80. doi: 10.1080/13607863.2011.605053, PMID: 21861766

[41] Dong Q Liu JJ Zheng RZ Dong YH Feng XM Li J. Obesity and depressive symptoms in the elderly: a survey in the rural area of Chizhou, Anhui province. Int J Geriatr Psychiatry. (2013) 28:227–32. doi: 10.1002/gps.3815, PMID: 22492613

[42] Kranjac AW Nie J Trevisan M Freudenheim JL. Depression and body mass index, differences by education: evidence from a population-based study of adult women in the U.S. Buffalo-Niagara region. Obes Res Clin Pract. (2017) 11:63–71. doi: 10.1016/j.orcp.2016.03.002, PMID: 27025915 PMC5035174

[43] Su Y Rao W D’Arcy C. Depression risk and body mass index among immigrants and non-immigrants in Canada: results from the Canadian community health surveys, 2010-2014. Soc Psychiatry Psychiatr Epidemiol. (2020) 55:1283–95. doi: 10.1007/s00127-020-01861-5, PMID: 32222875

[44] Luo X Ke X Li H Dai Q Zhang C Zheng W. Prevalence and risk factors for depression in outpatient departments of three general hospitals in China: a cross-sectional study. Int J Psychiatry Clin Pract. (2020) 24:88–95. doi: 10.1080/13651501.2019.1687723, PMID: 31718347

